# Academic Distinction in Medicine

**Published:** 1918-08-10

**Authors:** 


### The Hospital

The Workers' Newspaper of Administrative Medicine and Institutional Life, Administration, National Insurance and Health.

No. 1679, Vol. LXIV. SATURDAY, AUGUST 10, 1918. PRICE TWOPENCE

### ACADEMIC DISTINCTION IN MEDICINE.

Before us is a magazine cutting:—

"A striking testimony to the triumphs of women in the realm of art was given by Sir Edward Poynter in his address at the prize distribution of the Royal Academy in 1911, when he asked: 'Why do women beat men in the Academy school?'

"The answer was simple. The President found that, while the men were 'slack' and either did not know how to work or did not care, the women really were in earnest. An unprecedented succession of feminine triumphs had moved the President to make this comparison. Two of the four gold medals awarded by the Academy had gone to women. The gold medal for historical painting with the travelling studentship of £200, the coveted blue riband of the Academy, had been won by Miss ——; and the gold medal for landscape painting by Miss ——, in addition to which all the principal prizes in the painting . . . ." etc., etc.

Now, one might append to the above many compliments, but the instructive comment to make is that in all world history there has never yet been a woman painter of the first rank. Not for want of opportunity, clearly, because the drawing-master has long been prominent in the education of at any rate European women, while many famous male painters made enough mark to gain attention without any instruction at all. Of neither of the above-mentioned talented ladies, who distinguished themselves academically nearly a decade ago, has anything much been heard since; nor, similarly, is there on record an advance in mathematics due to Miss Philippa Fawcett, the long-ago conqueress of the Senior Wrangler. What I am trying to deduce from these phenomena is nothing at all ungallant, however, but just another exemplification of the truth set out for us in half a dozen ways by different famous men—that higher grades of human faculty will find their own education, are auto-educators; that instruction never made a rare talent, the lack of it never marred one; that breed is stronger than pasture, temperament everything, technique nothing . . . and so on in an ascending scale of earnest assertion. Now, in medicine we forget all about this—we worship the qualification, the scholarship, the medal.

We make the opportunity of access to plentiful clinical material—namely, a hospital appointment—dependent upon the holding of certain degrees, as though the original mind and the first-rate faculty must needs take kindly to a beaten path of study. And this, too, in youth, when scientific imagination is at its height. We actually distrust this very scientific imagination, talking as though great advances could come of institutes and team work and the activities of docile, conformable young men and women making circuit after circuit on the track of Baconian induction. Practically the only research prizes we offer are for set subjects. And all this in face of almost daily experience! For progress comes invariably from a rare individual faculty, expressing itself, pretty often, under hampering conditions. The Pasteur Institute, the Lister Institute, do plenty of good routine work, but they no more continue the name and fame of their founders than Richard Cromwell did the Protector's. Year after year the gold medallists get the gold medals, but the great medical *impasses* remain. Triennium after triennium the prizemen come to their half-positive, half-negative conclusions, the dust gathers on their theses, and they subside into money-getting. They are forgotten like the laboratory "research fellows," sure of promotion to a chair of pathology, who are set to see if such-and-such a German was right when he described a certain reaction as occurring in a high percentage of instances under certain conditions. But those whose work lives and fruits and grows, Sir Almroth Wright, Sir James Mackenzie, Sir Patrick Manson (two of these, you see, general practitioners, like Bodington was), started, not from a mechanical and superficial examination of the whole, not from any set examination at all, but by an original observation, a bit of insight; and with the peculiar tenacity of the born investigator they pursue their line to its end. The logicians have noted it, this creative beginning. But some of our eminent men, graceful scholars, diligent students, admirable figure-heads as they are, who have never had an original notion in their lives and never will have, the born text-book writers!—these personages must have read the logicians, yet they stand out against "insight" till the last possible moment. They call it prejudice, preconception. As though in this world, the redeeming feature of which is its ultimate favourability to truth and deadliness to error, the crank could masquerade as a discoverer for more than a year or two! Almost as if from moral considerations they present the investigator as made and not born; and, of course, satisfying of academic tests counts for much in the making.

Some achieve academic distinction, others get it by accretion. Here is one of the latter sort, our brisk and buoyant friend Dr. William Blower, worthy acolyte of The Humbug, whom you may be good enough to remember. He listened to that notability's lectures, thus taking in the elements of metropolitan medical teaching at second hand, kept on his right side in public, took little prizes against three or four competitors, took the M.R.C.P., read orotund papers to the Medical Society, quoting from no more inaccessible source than the *British Medical Journal*, and by virtue of his push came to succeed the old gentleman. In turn *he* retails the contents of London or Edinburgh or Dublin text-books to provincial students, thereby somehow qualifying for the Fellowship. Not so Dr. Leonard Williams, whose meritorious book (though I cannot agree with the reviewer that phrases like "grittily to greet the Resurrection" are good English) is in its fourth edition.

All of the above, such as it is, was written before the Royal College of Surgeons Fellowship examination was arraigned for its twenty-seven ploughed candidates out of thirty-four, and the Royal College of Physicians for poor judgment in bestowing distinction. Let these said protests, then, and the discussion ensuing upon them, act as a "spearhead" for what I have written. The present *régime* needs changing. Some people seem to think that that necessity may be obviated by abundant self-praise—an inexpensive means, but hardly a promising one. To others much taking of thought and trouble about reform appear necessary in this sphere of medicine, as in the rest. And as Truth notoriously loves difficult positions, I should look for her myself in the latter one. CADUCEUS.

